# Carbon Nanotubes Induce Metabolomic Profile Disturbances in Zebrafish: NMR-Based Metabolomics Platform

**DOI:** 10.3389/fmolb.2021.688827

**Published:** 2021-07-02

**Authors:** Raja Ganesan, Prabhakaran Vasantha-Srinivasan, Deepa Rani Sadhasivam, Raghunandhakumar Subramanian, Selvaraj Vimalraj, Ki Tae Suk

**Affiliations:** ^1^ Institute for Liver and Digestive Diseases, Hallym University, Chuncheon, Korea; ^2^ Department of Biological Sciences, Pusan National University, Busan, Korea; ^3^ Department of Pharmacology, Saveetha Dental College and Hospital, Saveetha Institute of Medical and Technical Sciences (SIMATS), Saveetha University, Chennai, India; ^4^ Department of Biotechnology, St. Peter's Institute of Higher Education and Research, Chennai, India; ^5^ PG & Research Department of Zoology, Ethiraj College for Women, Chennai, India; ^6^ Center for Biotechnology, Anna University, Chennai, India

**Keywords:** SWCNT, zebrafish, NMR metabolomics, high-throughput omics, metabolic pathways

## Abstract

The present study aims to investigate the metabolic effects of single-walled carbon nanotubes (SWCNT) on zebrafish (*Danio rerio*) using ^1^H nuclear magnetic resonance (^1^H-NMR) spectroscopy. However, there is no significant information available regarding the characterization of organic molecules, and metabolites with SWCNT exposure. Noninvasive biofluid methods have improved our understanding of SWCNT metabolism in zebrafish in recent years. Here, we used targeted metabolomics to quantify a set of metabolites within biological systems. SWCNT at various concentrations was given to zebrafish, and the metabolites were extracted using two immiscible solvent systems, methanol and chloroform. Metabolomics profiling was used in association with univariate and multivariate data analysis to determine metabolomic phenotyping. The metabolites, malate, oxalacetate, phenylaniline, taurine, sn-glycero-3-phosphate, glycine, N-acetyl mate, lactate, ATP, AMP, valine, pyruvate, ADP, serine, niacinamide are significantly impacted. The metabolism of amino acids, energy and nucleotides are influenced by SWCNT which might indicate a disturbance in metabolic reaction networks. In conclusion, using high-throughput analytical methods, we provide a perspective of metabolic impacts and the underlying associated metabolic pathways.

## Introduction

In the past few decades, the carbon nanotubes (CNT) have gained a great attention in biomedical applications that are observed as pipe-shaped atomic layers of carbon particles. It is made up of layer-by-layer orientation of graphite sheets (Cheung et al., 2000; Wang et al., 2021; Zhou et al., 2021). Single-walled carbon nanotubes (SWCNT) and multi-walled carbon nanotubes (MWCNT) have recently applied in lung cancer therapy as a novel drug delivery system, with topographies ranging from 10 to 1,000 nm in size and length ranging from 0.5 to 20 nm. Inner hollow space, electrostatic potential, ultralightweight, drug encapsulating ability, and cellular penetration or transmission abilities are characteristics of CNT (Sahoo et al., 2011; Wu et al., 2011). CNT, polymer nanotubes, and surface altered-CNT have used in clinical applications over two decades (Hirsch, 2002).

The hydrophobic nature of CNT, as well as poor biocompatibility in solvent media, is its drawbacks. Furthermore, surface changes in materials, such as chemical alterations in CNT, may improve solubility in an aqueous environment (Yu et al., 2006; Cho et al., 2015). When working on the surface of CNT, the adsorption, carboxylation, amination, esterification, and surface polymer coating have all are considered (Bhandavat et al., 2013; Yao et al., 2017). The π−π interface between tubes (0.5–2 eV/nm), H-bonding, and van der Waals forces are commonly occurred in the aquatic dispersant. According to previous publications in aquatic cytotoxicity investigations, biocompatible solvent materials of Tween 80, polyethylene glycol (PEG), and dimethyl sulfoxide (DMSO) are reported to use CNT dispersion (Afraz et al., 2014; Chopdey et al., 2015; Carranza et al., 2016). However, water is used as a solvent because of its low cost and greater potential for green chemistry.

The doxorubicin (DOX) and folic acid (FA) coated CNT are achieved higher anti-proliferative effects. The cytotoxicity is found to be associated with DOX and FA in MCF-7 cells (Katwa et al., 2012; Lu et al., 2012; Chall et al., 2021). CNT size, length, sub-lethal concentration (LC_50_), the path of inhalations, and solvent materials played a lead role in toxicological responses (Han et al., 2010; Ronzani et al., 2012). Recently, MWCNT has employed in animals such as zebrafish, copepod (i.e., *Tigriopus japonicas*), frogs (i.e., *Xenopus laevis*), rat, and mice to profile the toxicological responses(Allegri et al., 2016; Girardi et al., 2017; Qi et al., 2017). In *in vivo* analytical and molecular mechanisms, liver, spleen, and kidney are examined with CNT exposures which could deliver deep knowledge of toxicity levels (Pauluhn, 2010; Kasai et al., 2016).

MWCNT showed impact on apoptosis, DNA damage, oxidative stress, and inflammation, as well as enzyme alteration, protein modification, and gene expression (Ma-Hock et al., 2009; Mercer et al., 2013). Hydrolase and albumin modifications as well as metabolic reactions have studied at the proteome level with CNT exposures (Dong and Ma, 2017; Poulsen et al., 2017; Lee et al., 2018). Free radicals and reactive oxygen species (ROS) are also activated by CNT (O_2_^−^) in dose dependent manner (Shvedova et al., 2003; Monteiro-Riviere et al., 2005; Yuan et al., 2012). Hence, it is speculated that CNT can influence metabolic profile disruption.

To test CNT effect on metabolic profile, zebrafish (*Danio rerio*) is used as a model organism. Zebrafish is a tiny tropical freshwater fish. Zebrafish are an important vertebrate model organism that is frequently used in toxicological research due to their ease of maintenance in laboratory conditions. (Garcia et al., 2016).

Because of the low cost per sample, lack of requirement for derivatization, and the ability to measure and identify both known and unknown compounds, NMR spectroscopy is the good technology for metabolomics investigations with therapeutic relevance. (Cappello, 2020). The NMR spectroscopy technique describes the chemical input, biosynthetic intermediates, and end products of cellular activity. ^1^H-edited NMR-based metabolomics profiling in zebrafish has several distinct advantages in terms of quantitative and qualitative metabolites (Fessenden, 2016; Puchades-Carrasco and Pineda-Lucena, 2017; Raja et al., 2020a; Raja et al., 2020b; Raja et al., 2021). Metabolomics analysis allows for more accurate correlations between cellular transition and biochemical pathways. Metabolomics provides a high-throughput global metabolite analysis that is arguably more of an essential platform in an omics science (Blow, 2008; Guijas et al., 2018; Raja et al., 2020a; Raja et al., 2020b) (Dumas et al., 2006). In a separate metabolomics experiments, we assessed the accuracy of metabolic compounds under different conditions. Here, we examined the metabolic phenotypic expression and biochemical reaction that would lead to the identification of new therapeutic metabolites. The present study may provide a perspective of metabolic profile and the underlying associated metabolic pathways influenced by SWCNT in zebrafish model.

## Methods and Materials

### Chemicals and Materials

Methanol (CH_3_OH), chloroform (CHCl_3_), sulfuric acid (H_2_SO_4_) and nitric acid (HNO_3_) were obtained from Carlo Reactifs (SDS, France). SWCNT (Cat No: 775533; CAS No: 308068-56-6; >95% of carbon as SWCNT), and sodium salt of 3-(trimethylsilyl) propionic-2,2,3,3-d_4_ acid (TSP-d_4_) were purchased from Sigma-Aldrich, United States. According to datasheet, carbon content (≥95%), color, black; appearance, the powder was confirmed by Thermogravimetric (TGA) investigation. Fish food was obtained from Tera (Melle, Germany). Deuterium water (D_2_O: 99.8% purity) was obtained from Cambridge Isotope Laboratories, United States. Food was purchased from Tera (Melle, Germany).

### Zebrafish and Sample Collection

Zebrafish (*Danio rerio*; average weight, 0.5–0.9 g; average dimension, 3.6–4.2 cm) were obtained from a local dealer. The age ranges from 5 to 6 months. Zebrafish were maintained for more than two weeks in glass tanks, which contained a fresh 60 L dechlorinated water. Fish were fed 1.0% body weight twice daily with commercial food. All animals were standardized at 26.0 ± 1°C and maintained on 14:10 h light/dark for a reproductive cycle. All examinations were carried out with various dosages of SWCNT (control, 0; 10, 50, and 100 mg/L) for 72 h. Each experimental exposure contains eight fish (*n* = 8, totally 32) which contains equally in a treated tank. These concentrations were determined based on the previous publications (Cheng et al., 2009; Lee et al., 2015; Lee et al., 2016). The feeding was stopped. After 72 h, all fish was cleaned in additional water, and frozen in liquid nitrogen. Then, the homogenization is done to make a fine powder by mortar and pestle. Finally, fine powder of samples was collected in test tubes. All procedures were carried out on fish were approved by the Institutional Animal Care and Use Committee (IACUC) of the Anna University Center for Biotechnology.

### Sample Preparation and Metabolite Extraction

The metabolites were extracted according to previous reports (Bligh and Dyer, 1959; Martin et al., 2007). Briefly, at 4°C, 1,600 µL of 1:1 ratio of ice-cold solvent methanol and chloroform was added to powdered fish. After that, 15 min of sonication and 5 min of vigorous vortexing was done. Then, 1,400 µL of ice water was added to make biphasic conditions. After homogenization, at 4°C, the mixture was centrifuged at 291.79 g (3,000 rpm) for 10 min. Next, the upper part of the aqueous phase was collected and the samples were lyophilized. 700 µL of 99.9% deuterated phosphate buffer (0.2 M, pH 7.0) in heavy water with TSP-d4 was mixed with aqueous samples. Finally, 700 µL of dissolved sample mixture was transferred to 5 mm NMR tubes.

### Acquisition

For temperature equilibration, the samples were kept for 10 min inside the NMR probe. NMR spectra were recorded on a 600 MHz spectrometer using one NMR probe. A water-suppressed t_2_-edited CPMG pulse sequence (RD-90°-[τ-180°-τ]n) was applied to the visualization of small molecules/metabolites. With relation to solvent peaks (TSP at 0 ppm), ^1^H-NMR chemical shifts were described in ppm (δ). The chemical shifts from −2 to 14 ppm were covered and the multiplicity stated as s, singlet; d, doublet; t, triplet; q, quartet; and m, multiplet. Heavy water acts as field frequency locking. The acquisition time, 3.0 s; relaxation delay, 1.0 s; width, 9,615.6 Hz; and 128 scans were acquired from each spectrum. Total time per sample was taken for 10 min.

### Data Processing

All Fourier transform spectra were manually corrected for phase and baseline distortions. TSP calibration at 0 ppm was processed. Mnova software (Mestrelab Research, Santiago de Compostella, Spain) was used to visualize, process the 1D NMR data. To avoid statistical error, the water domain (δ 4.7–4.9 ppm) was removed. δ 0–10 ppm was uniformly divided as buckets that have 0.001 ppm. Further, binned data were exported to MetaboAnalyst software (v 5.0) for multivariate statistical analysis. Initially, to eradicate outlier data, principal component analysis (PCA) was analyzed (data not shown). Using supervised techniques, particle least squares discriminant analysis (PLS-DA) and orthogonal PLS-DA (OPLS-DA) were applied to NMR data. From score data, each point represents a ^1^H- spectral data.

### Data Handling and Statistical Analysis

The data normalization (to avoid concentration differences among metabolites) and validation for score plot analysis was accomplished using the Pareto scaling algorithm, which measured each variable using the square root of its standard deviations. MetaboAnalyst 5.0 program (https://www.metaboanalyst.ca/) was used to analyze all of the samples. The newly aligned and filtered metabolite lists were then processed. The metabolic expression, metabolic networking, and biological interpretation were determined after a logarithmic transformation. GraphPad Prism eight software was used to perform statistical analysis. Numerical values were delivered as the mean ± SD. Two-way analysis of variance (ANOVA) has been used to examine multiple subjects. The significant changes of metabolites were recognized by *p* < 0.05.

## Results

### High-Throughput and Highly Reproducible NMR-Based Platform

**FIGURE 1:**
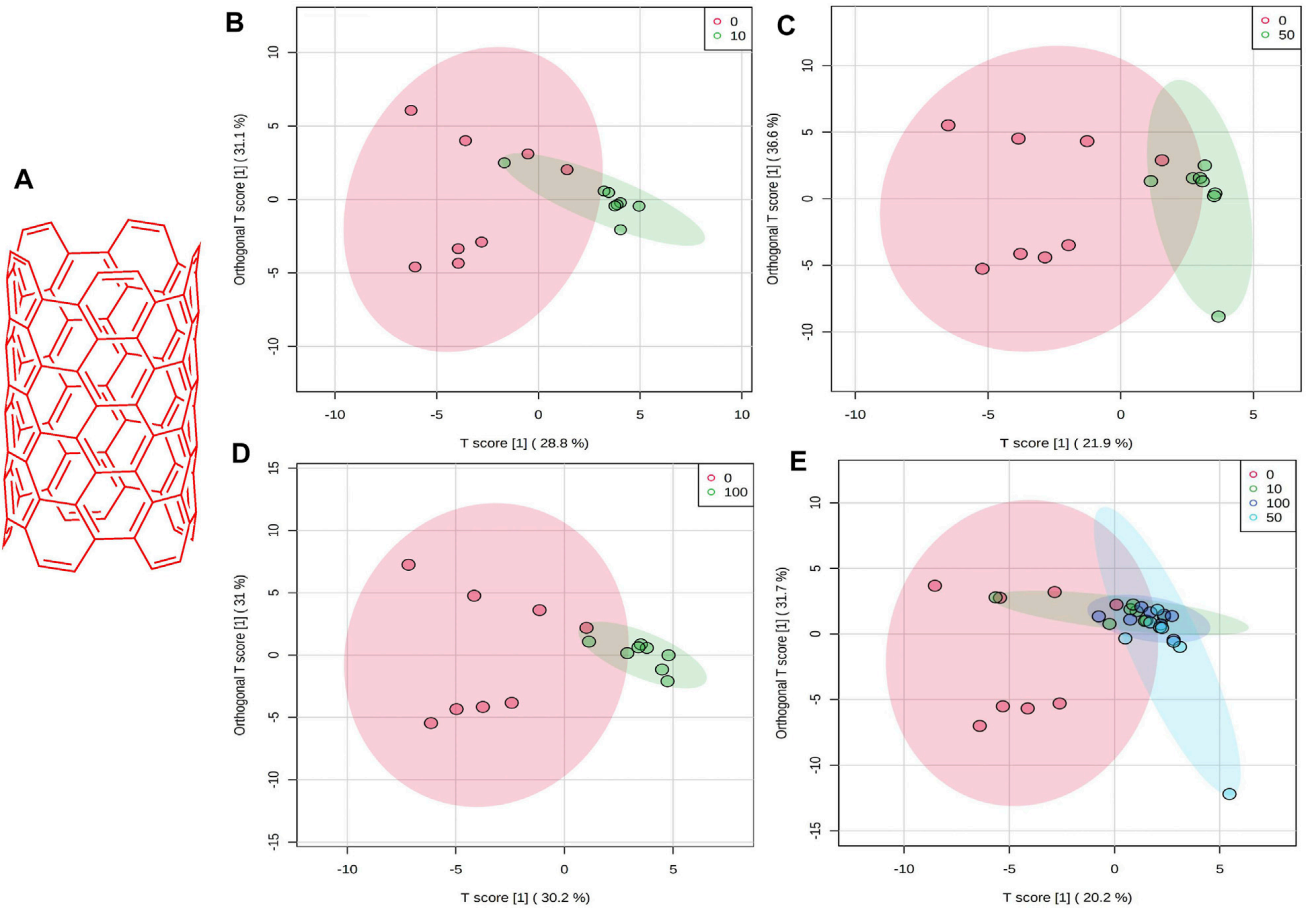
**(A)** Schematic chemical structure of SWCNT. An OPLS-DA predictive model of zebrafish metabolite profiles after SWCNTs exposure. **(B)** 0 vs 10 mg/L, 59.9%, Red dot, 0 mg/L; Green dot, 10 mg/L. **(C)** 0 vs 50 mg/L,58.5%, Red dot, 0 mg/L; Green dot, 50 mg/L. **(D)** 0 vs 100 mg/L, 61.2%, Red dot, 0 mg/L; Green dot, 100 mg/L. **(E)** OPLS-DA score plot on normalized metabolite concentrations of 0 vs 10, 50, 100 mg/L. PC can be found in 51.9% of discriminations. Red dot, 0 mg/L; Green dot, 10 mg/L; light blue, 50 mg/L; and dark blue, 100 mg/L.

The chemical structure of SWCNT is shown in Figure 1A. The percentage (%) values, rules, and relevant *p*-values are mentioned in Table 1. Fold variations (FC > 1.0, increased; FC < 1.0, decreased) are taken into account when determining metabolite levels. Several small molecules, and metabolites (amino acids, polyamines, nucleosides, fatty acids, phosphates, carbohydrates, organic molecules, and other organic mixtures) were observed in spectral chemical changes. The phosphate buffer ^1^H-resonance at 0 ppm was used as a reference peak. Each metabolite or molecule have its hydrogen-1 nuclei (e.g., −CH, −CH_2_, and −CH_3_) profiled and quantified. Mnova software was used to organize the spectral data. Before and after normalization with SWCNT exposed metabolites, Pareto Scaling-based Kernel density plots and Box plots analysis are shown in Supplementary Figure S1. The density plots are based on all samples, while the box plots represent the most quantified metabolites.

**TABLE 1:** The description of the quantified metabolites in metabolomic fingerprint associated with decompensatory state in SWCNT with three scenarios.

No	Name	0 vs 10			0 vs 50			0 vs 100		
		% Cal	Increase or decreases	*p* Values	% Cal	Increase or decreases	*p* Values	% Cal	Increase or decreases	*p* Values
1	2-Oxoglutarate	73.70449	Increase	0.092747	10.7,617,737	Increase	0.389,275	74.77,899,388	Increase	0.05272
2	ADP	162.8223	Increase	0.001449	314.7,813,657	Increase	0.097672	513.2,112,066	Increase	0.020734
3	AMP	198.2004	Increase	0.096359	219.947,296	Increase	0.062638	344.1,798,478	Increase	0.029183
4	ATP	7.437,242	Increase	0.431,091	158.8,837,666	Increase	0.046208	81.07,693,581	Increase	0.057041
5	Acetate	-16.4859	Decrease	0.421,167	-54.00823,801	Decrease	0.243,334	-36.91,082,151	Decrease	0.317,866
6	Alanine	15.59503	Increase	0.365,929	-14.58,162,808	Decrease	0.374,807	-2.684,656,139	Decrease	0.475,424
7	Anserine	-34.607	Decrease	0.130,985	-23.22,183,101	Decrease	0.234,101	-31.87,973,584	Decrease	0.135,223
8	Arginine	-9.62773	Decrease	0.395,644	-41.06,335,018	Decrease	0.092192	-9.285,285,347	Decrease	0.389,061
9	Asparagine	104.8953	Increase	0.001219	45.50,601,736	Increase	0.085467	109.4,722,721	Increase	0.003295
10	Aspartate	60.73794	Increase	0.129,267	17.19,689,515	Increase	0.350,938	61.71,678,763	Increase	0.088883
11	Betaine	-74.2478	Decrease	0.030403	-81.08,560,073	Decrease	0.017013	-87.29,947,554	Decrease	0.011894
12	Choline	-3.72769	Decrease	0.441,084	-15.94,533,167	Decrease	0.257,837	6.133,147,486	Increase	0.408,377
13	Citrate	76.54633	Increase	0.084544	19.49,283,991	Increase	0.292,368	40.0717,263	Increase	0.161,152
14	Creatine	40.32183	Increase	0.055934	42.31,849,693	Increase	0.030331	46.51,900,655	Increase	0.016909
15	Creatine phosphate	13.98336	Increase	0.380,374	-4.797,583,356	Decrease	0.454,801	-2.90,649,314	Decrease	0.471,668
16	Creatinine	188.339	Increase	0.00438	79.80,155,794	Increase	0.110,077	201.647,253	Increase	0.00026
17	Dimethylamine	-97.8965	Decrease	0.011613	-88.62,817,939	Decrease	0.020103	-96.41,436,763	Decrease	0.012514
18	Ethanol	-77.5631	Decrease	0.008482	-67.1,337,193	Decrease	0.022809	-83.83,115,472	Decrease	0.005507
19	Formate	25.81097	Increase	0.222,801	-24.04,594,779	Decrease	0.17447	-31.44,758,524	Decrease	0.089225
20	Fucose	144.9785	Increase	0.040645	34.82,528,867	Increase	0.274,614	136.3,851,508	Increase	0.023485
21	Fumarate	-98.3009	Decrease	0.024533	-90.61,049,235	Decrease	0.035229	-98.65,290,734	Decrease	0.024179
22	GTP	-81.3516	Decrease	0.020048	-39.23,261,294	Decrease	0.246,862	-84.14,978,973	Decrease	0.017079
23	Glucose	24.36722	Increase	0.257,163	36.4,047,155	Increase	0.2598	-10.7,777,396	Decrease	0.381,518
24	Glutamate	38.88837	Increase	0.219,095	-15.81,553,187	Decrease	0.356,268	38.5,708,216	Increase	0.185,623
25	Glutamine	24.25334	Increase	0.26184	27.57,064,527	Increase	0.211,586	52.81,697,604	Increase	0.111,108
26	Glutathione	-25.4743	Decrease	0.266,799	10.94,984,271	Increase	0.437,511	-11.27,347,073	Decrease	0.395,667
27	Glycerol	-23.5594	Decrease	0.324,996	-50.34,917,191	Decrease	0.150,181	-46.73,837,336	Decrease	0.156,496
28	Glycine	133.4323	Increase	0.003889	105.1,663,781	Increase	0.037436	129.3,957,885	Increase	0.004141
29	Histidine	74.85256	Increase	0.024275	56.62,485,047	Increase	0.120,098	75.82,464,931	Increase	0.015855
30	Hypoxanthine	-35.6985	Decrease	0.134,947	-45.75,566,132	Decrease	0.074832	-54.45,315,064	Decrease	0.0401
31	Inosine	12.23891	Increase	0.353,662	32.79,363,508	Increase	0.222,122	-19.10,798,266	Decrease	0.255,749
32	Isoleucine	-28.8215	Decrease	0.267,945	-71.67,649,692	Decrease	0.026533	-60.78,558,236	Decrease	0.046238
33	Lactate	114.2764	Increase	0.023645	83.76,939,699	Increase	0.08791	87.79,790,125	Increase	0.063472
34	Leucine	-9.10471	Decrease	0.450,083	-74.6,175,899	Decrease	0.103,589	-51.55,430,254	Decrease	0.188,734
35	Lysine	-23.6233	Decrease	0.365,803	-69.53,369,684	Decrease	0.102,491	-56.27,603,841	Decrease	0.150,371
36	Malate	136.4495	Increase	0.014655	93.6,135,441	Increase	0.04656	270.6,447,781	Increase	0.003909
37	Methionine	-30.2841	Decrease	0.297,722	-75.18,860,852	Decrease	0.050906	-67.01,984,485	Decrease	0.069927
38	N-Acetylaspartate	-22.321	Decrease	0.191,535	-17.51,070,044	Decrease	0.241,316	-27.39,036,923	Decrease	0.105,742
39	N-Acetylglutamate	-98.1887	Decrease	0.028904	-81.39,713,565	Decrease	0.065271	-99.14,360,319	Decrease	0.027846
40	NAD+	40.94824	Increase	0.174,951	-42.46,688,684	Decrease	0.108,032	-9.34,639,557	Decrease	0.414,604
41	NADPH	-86.1827	Decrease	0.023109	-70.85,400,513	Decrease	0.057615	-87.19,187,358	Decrease	0.022166
42	Niacinamide	30.50126	Increase	0.084566	90.58,143,772	Increase	0.006595	79.05,040,869	Increase	0.004627
43	O-Phosphocholine	-88.2178	Decrease	0.0245	-60.95,103,293	Decrease	0.126,429	-92.31,146,815	Decrease	0.019682
44	O-Phosphoethanolamine	81.26837	Increase	0.005801	29.64,934,434	Increase	0.152,594	93.51,890,578	Increase	0.004082
45	Oxalacetate	246.0446	Increase	0.001964	213.6,985,716	Increase	0.011506	413.511,089	Increase	3.17E-05
46	Phenylalanine	-74.0998	Decrease	0.031956	-90.1,019,858	Decrease	0.008298	-88.17,690,096	Decrease	0.009291
47	Proline	82.14609	Increase	0.094111	-13.42,018,188	Decrease	0.38575	46.6,589,559	Increase	0.172,693
48	Pyruvate	103.5242	Increase	0.031163	103.6,141,295	Increase	0.036117	127.1,389,676	Increase	0.000509
49	Serine	33.88681	Increase	0.245,025	10.72,826,148	Increase	0.400,198	19.90,400,252	Increase	0.304,549
50	Succinate	-54.6477	Decrease	0.148,816	-59.82,893,254	Decrease	0.127,362	-66.25,893,766	Decrease	0.105,893
51	Taurine	178.7991	Increase	0.004197	152.5,804,078	Increase	0.010755	216.5,095,771	Increase	0.000156
52	Threonine	-47.4423	Decrease	0.067747	27.69,784,522	Increase	0.35361	-29.84,662,431	Decrease	0.171,608
53	Trimethylamine	8.60628	Increase	0.380,823	26.0161,051	Increase	0.147,961	13.02,893,349	Increase	0.293,997
54	Trimethylamine N-oxide	-85.3013	Decrease	0.072336	-38.30,154,071	Decrease	0.296,117	-80.02,162,641	Decrease	0.085984
55	Tryptophan	-25.9805	Decrease	0.31299	-62.68,571,426	Decrease	0.054649	-59.2,106,272	Decrease	0.063837
56	Tyrosine	-58.317	Decrease	0.070382	170.2,144,897	Increase	0.254,126	-72.94,281,412	Decrease	0.022625
57	Uridine	-63.7642	Decrease	0.09481	-55.92,116,852	Decrease	0.13265	-73.84,546,235	Decrease	0.065303
58	Valine	-40.1231	Decrease	0.153,864	-74.18,575,041	Decrease	0.011754	-57.83,807,623	Decrease	0.035354
59	Xanthine	-25.3289	Decrease	0.346,071	80.03,241,069	Increase	0.210,728	-33.66,130,094	Decrease	0.309,344
60	Myo-inositol	-47.6804	Decrease	0.1441	-65.48,314,175	Decrease	0.072438	-51.37,657,254	Decrease	0.12524
61	sn-Glycero-3-phosphocholine	-82.4237	Decrease	0.033543	-88.18,738,357	Decrease	0.025833	-80.9,428,004	Decrease	0.035817

Note and abbreviations: 0, control; 10 mg; 50 mg; 100 mg; % Cal, percentage calculations.

### Quantification, Assessment, and Pattern Recognition Analysis

There was a strong distinction between the four classes (i.e., 0, 10, 50, and 100 mg/L), suggesting that zebrafish has a unique metabolic profile. The possible metabolites (FDR 0.05, FC 1 and FC > 1) are highlighted in the volcano map. A univariate OPLS-DA score plot of SWCNT-applied samples shows that they are widely separated from each other and starving fish (Figures 1B–D) (control zebrafish). The metabolic differences in 10 mg/L, 50 mg/L, and 100 mg/L were found to be 59.9%, 58.5 percent, and 61.2 percent, respectively. In addition, the OPLS-DA score plot of SWCNT in zebrafish is shown in Figure 1E. The score plots are demonstrated in the 95% confidence region.

**FIGURE 2:**
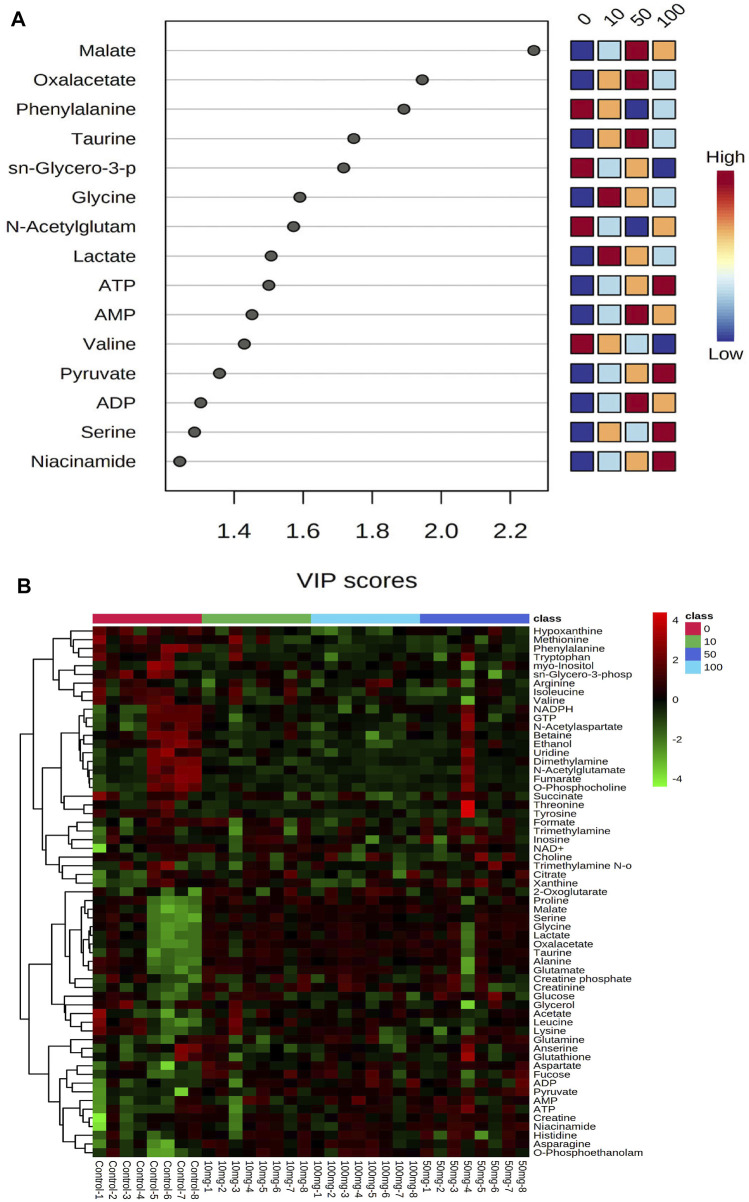
Interactive heatmap analysis with metabolites or small molecules dataset visualization in Zebrafish after SWCNT exposures. The rows and columns display metabolites and the samples, respectively. The green and red colors indicate down- and upregulated metabolites, respectively, in fish fluid samples. The brightness of each color corresponds to the alteration magnitude compared with the average value. The classes of 0, 10, 50, and 100 indicate control, 0 mg/L, 10 mg/ml, 50 mg/ml, and 100 mg/ml, respectively.

PLS-DA scores were used to select the candidate quantified metabolites with a variable importance of projection (VIP) score >1 (Song et al., 2011). Fifteen metabolites, including malate, oxalacetate, phenylalanine, taurine, sn-glycero-3-phosphate, glycine, N-acetylglutamate, lactate, ATP, AMP, valine, pyruvate, ADP, serine, and niacinamide, are differentiating the metabolic functions according to VIP >1 score analysis (Figure 2A). The heatmap in Figure 2B illustrates the phenotypic delivery of quantified metabolites. The average intensity of variation in the respective sets is represented by painted bars. CNT induces a net increase or decrease in the metabolite amount, as shown by the red and green colors.

As shown in Supplementary Figure S2A, the metabolic correlation map was plotted. The analysis focused on the metabolic expression caused by SWCNT in zebrafish compared to control zebrafish. The metabolite expression from SWCNT-treated zebrafish is shown in the row, while the metabolite expression from control zebrafish is shown in the column. The metabolites whose regulation had basically decreased were shown in green, while those whose regulation had increased were shown in red. This data, when combined with the dendrogram of various leveled bunch investigations, provides a worldwide perspective of metabolite changes in SWCNT-affected zebrafish metabolisms. A significant analysis of metabolites (SAM plot) has been done in Supplementary Figure S2B and screened 12 metabolites as candidate metabolites. The relative expression of metabolites is shown in Supplementary Figure S2C. The cumulative metabolic discrimination is 52.9%. The score plot parameters that *R* ^2^ (0.8: 80%) and Q^2^ (0.4: 40%) revealed satisfactory goodness of fit and goodness prediction, respectively (Supplementary Figure S3A,B). Q^2^ indicates that a four-component model is the best (marked with a red star). *R* ^2^ and Q^2^ values were indicating near 1 that was considered as robust score plots. The evidence of *R* ^2^ and Q^2^ values from score plots indicate that the metabolic disconcertion was found in fish by SWCNT exposures.

### Metabolic Phenotypic Changes and Expression

Adenosine phosphates are involved in energy hydrolysis in cells (ATP to ADP to AMP), and these phosphates serve as a caption of energy yield. AMP, ADP, and ATP are increased in low to high concentrations. These changes also perplexed the ratio of total adenosine phosphate groups, which could disrupt the zebrafish's equilibrium between energy production and ATP consumption. The AMP-activated protein kinase (AMPK) pathway is activated by metabolic changes in adenosine phosphate (Duft et al., 2017; Simpson-Lavy et al., 2017). In cellular environments, demanding ATP production can occur.

Anaerobic conditions can develop in zebrafish as a result of adenosine phosphate degradation. The metabolic disturbance caused by SWCNT exposures, which involves increased ADP, ATP, and decreased glucose, disrupts ATP intake in cellular environments, which serves as cellular energy. When the zebrafish was given large doses of SWCNT, the metabolic chemical reaction took place primarily via the oxygen-dependent pathway. ATP, ADP's primary metabolite, is formed during purine metabolism. As a result, these findings indicate that SWCNT disrupts purine metabolism.

**FIGURE 3:**
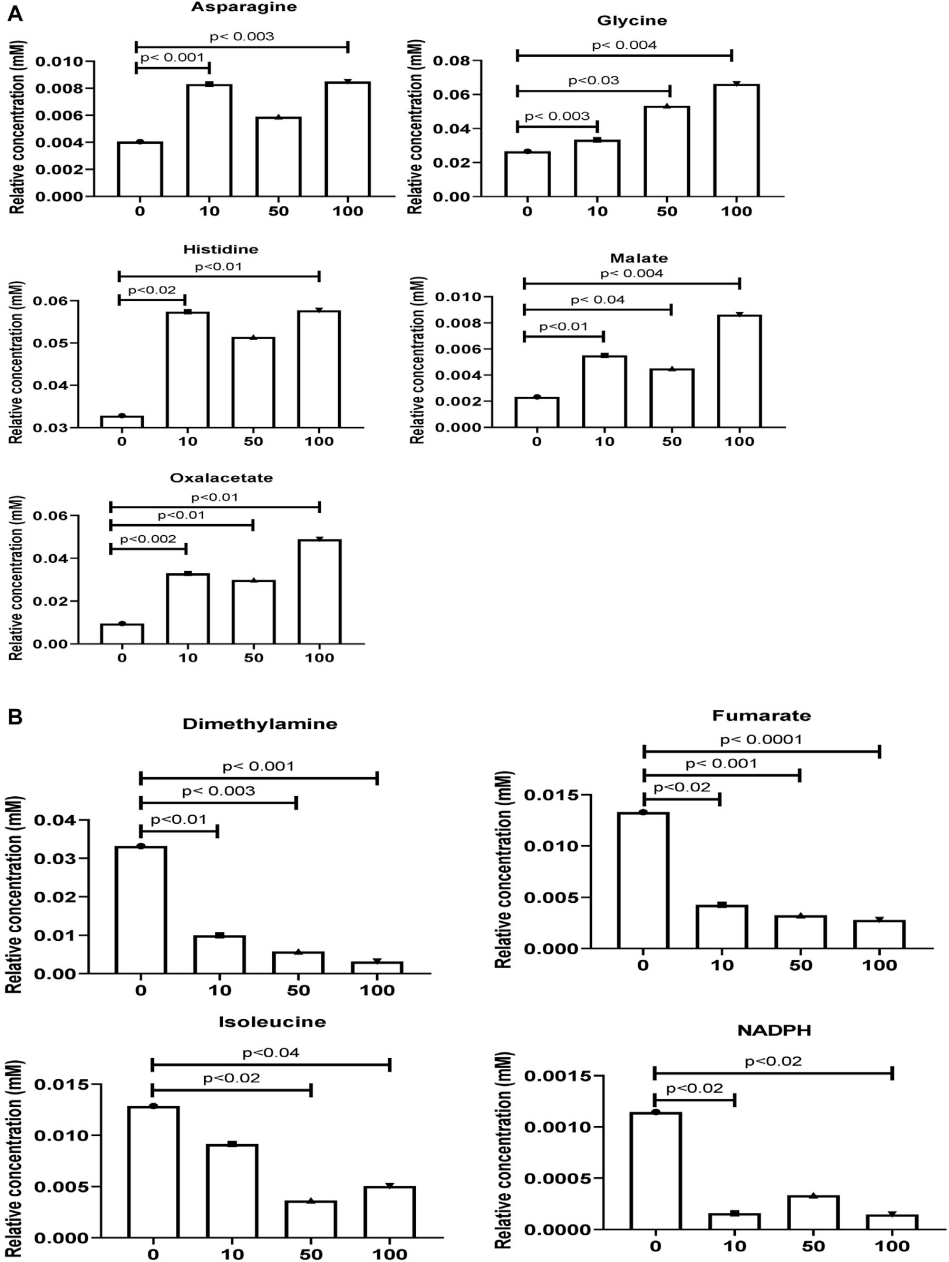
Comparison of the normalized relative intensities of the potential metabolites between control and SWCNT applied zebrafish **(A)** The employed strategy of the automated assignment of metabolites, asparagine, glycine, histidine, malate, and an oxalacetate with significantly increased concentration from control samples and differences are shown. **(B)** The significantly decreased metabolites, dimethylamine, fumarate, isoleucine, and NADPH has been listed. *t*-test analysis; *p* < 0.05, *p* < 0.01, *p* < 0.001 are considered significant metabolites. Data show mean ± SEM.

As shown in Figures 3A,B, the original concentration range of an asparagine, glycine, histidine, malate, and oxalacetate were significantly increased by SWCNT exposures in zebrafish. Reversibly, it is important to note that the dimethylamine, fumarate, isoleucine, and NADPH levels were significantly decreased with SWCNT exposures. Thus, different metabolic fractions can affect their biological functions.

**FIGURE 4:**
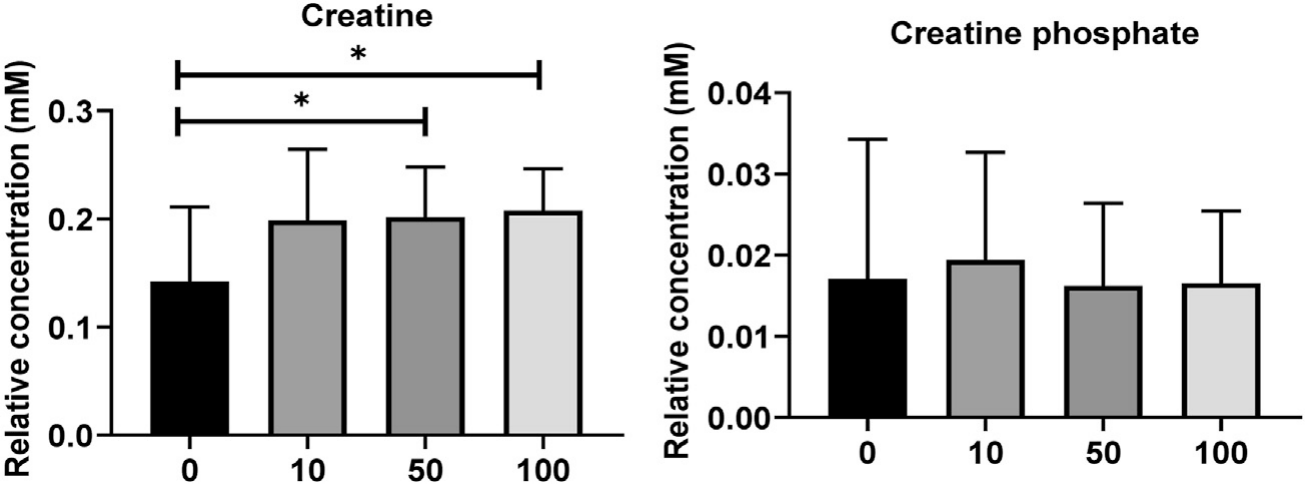
**(A)** Level of significantly dysregulated creatine and creatine phosphate in zebrafish. **(B)** The top 15 most metabolic discriminating metabolites between Control and SWCNT. VIP metabolome identified from fish relative to the controls after the SWCNT treatment. The major contributing metabolites are identified using PLS-DA algorithm. The right heatmap shows the concentration of the metabolites. The middle part shows the VIP scores. The left part lists the significant difference between metabolites.

Metabolites of NAD-utilizing reactions such as oxaloacetate are found to be significantly increased. The NAD involved oxidation in TCA cycle intermediates such as acetyl CoA, alpha-ketoglutarate, and succinyl-CoA can perplex the ATP energy production and metabolic pathway process. Pyruvate, oxaloacetate, NADPH, and NAD + have significantly increased which may lead to an intra and extracellular oxidative chemical reaction. Furthermore, upregulated NADPH/NAD + ratio has been perplexed and lead to ROS development which could affect the oxidative chemical reaction in lipid metabolisms, glycolysis, and galactose metabolism. As shown in Figure 4, the creatine and creatine phosphate has been increased. These two metabolites act as reserve high-energy phosphates in muscles.

According to the latest updates, carbon nanoparticles and carbon nanotubes (CNTs) uptake causes oxidative stress. The internalization of nanoparticles and their aggregation in cells have been explored (Li et al., 2011; Chiesa et al., 2018). The accumulation of titanium nanomaterials larger than 100 nm in the inner and outer membranes, as well as the intracellular environment (i.e., cytosolic), has been shown to cause stress in mitochondrial and cytoplasmic environments, resulting in autophagy (Bu et al., 2010; Tang et al., 2010).

**FIGURE 5:**
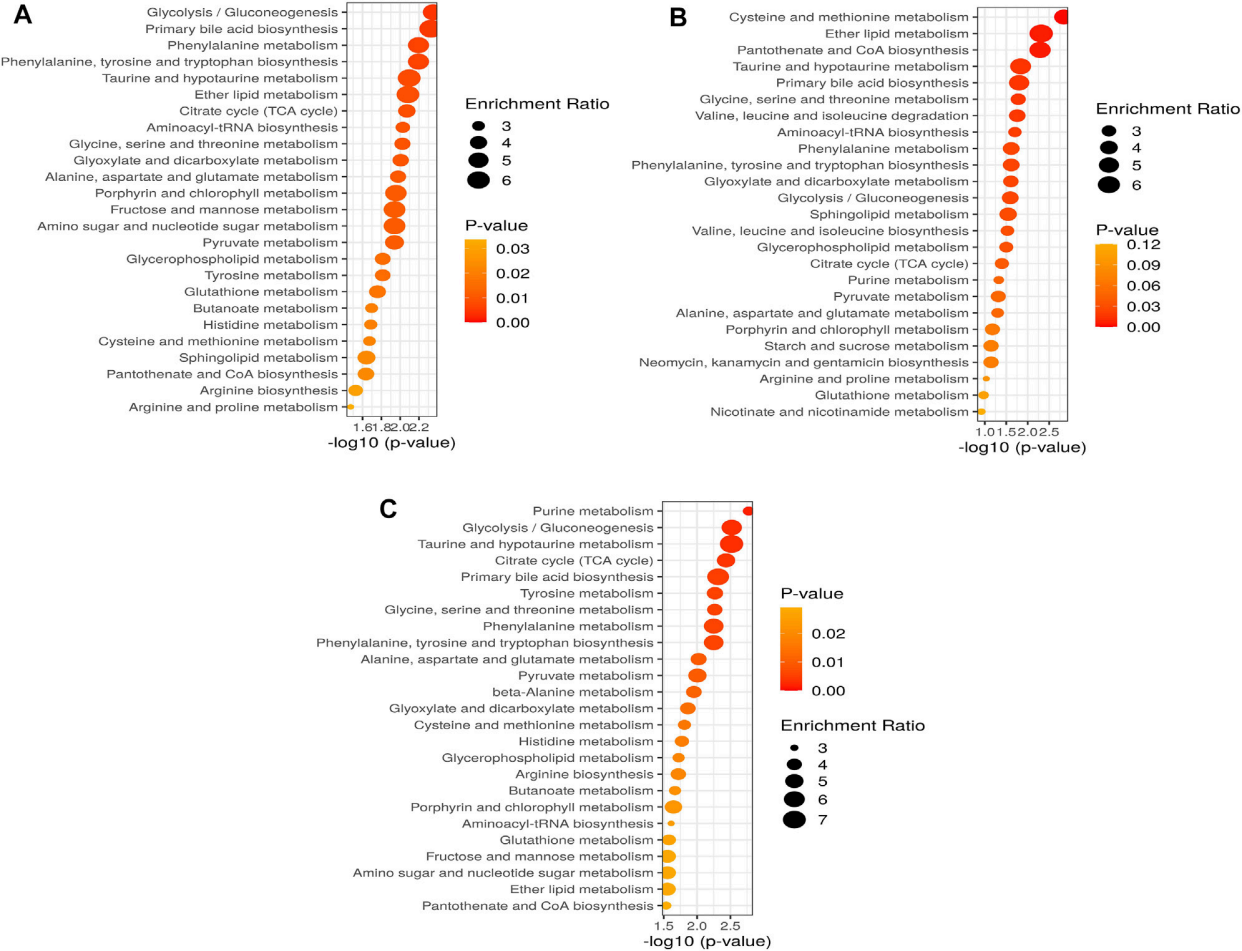
Altered metabolic pathways and performance of the automated assignment in zebrafish in **(A)** 10 mg/L; **(B)** 50 mg/L; **(C)** 100 mg/L. The differential metabolites identified by all studies under review were enriched into pathways using MetaboAnalyst software (version 5.0; www.metaboanalyst.ca). Pathways where metabolite sets are ranked according to *p*-value with hatched lines indicating Holm *p*-value threshold.

As shown in Figures 5A–C and Supplementary Tables S1–3, the metabolic pathways in 10 mg/L: glycolysis/gluconeogenesis (26/5), primary bile acid biosynthesis (46/2), phenylalanine metabolism (10/2), phenylalanine, tyrosine, and tryptophan biosynthesis (4/2), taurine and hypotaurine metabolism (8/1), ether lipid metabolism (20/1), citrate cycle (20/6); 50 mg/L: cysteine and methionine metabolism (33/3), ether lipid metabolism (20/1), pantothenate and CoA biosynthesis (19/2), taurine and hypotaurine metabolism (8/1), primary bile acid biosynthesis (46/2), glycine, serine and threonine metabolism (33/7); and 100 mg/L: purine metabolism (65/8), glycolysis/gluconeogenesis (26/5), taurine and hypotaurine metabolism (8/1), citrate cycle (20/6), primary bile acid biosynthesis (46/2) were significantly affected by SWCNT. The values after each metabolic pathway that explained the hits (right) and totals (left) represent the number of metabolites present and total metabolites involved in that metabolism, respectively. To find the pathways phenotypic expression, the metabolites set enrichment analysis (MSEA) has been performed to find the affected highly disturbances in every metabolic pathway.

## Dicussion

This is the first study to use a zebrafish model to evaluate SWCNT's effects on metabolites and determine if those metabolites are upregulated or downregulated in their metabolism. The density plots that before normalization (left side), after normalization (right side) are delivered based on all the metabolites datasets. These box plots defined the distribution of each variable or metabolite concentrations (before and after normalization). According to *pareto-scaling,* normalization, and *trans*-formation, the metabolomic dataset was deferred. Kernel density estimation, which used to estimate the probability density functions. This analysis may yield the practical applications of probability distributions.

A univariate and multivariate analyses of OPLS-DA score plot was constructed, delivering relative clustering positions with SWCNT treatments. Among these, four groupings (Control vs. 10, 50, and 100 mg/L of SWCNT) exhibited clear separation between the groups. The *R* ^2^ and Q^2^ values are reported, which decreases as the score plot becomes robust (i.e., R^2^-Q^2^). We first analysed the metabolites and associated metabolic pathways in zebrafish metabolisms with SWCNT exposures. The detection of top fifteen metabolites such as malate, oxalacetate, phenylaniline, taurine, sn-glycero-3-phosphate, glycine, N-acetyl mate, lactate, AMP, ADP, ATP, valine, pyruvate, serine, and niacinamide has been significantly impacted by SWCNT exposures. Here, an asparagine, glycine, histidine, malate, and oxalacetate are increased from control zebrafish. The TCA metabolites, malate, fumarate and oxalacetate contribute to the production of energy (Bruzzone et al., 2020).

The amino acid of histidine is required for protein biogenesis. These metabolites were then selected as prognostic biomarkers through differential expression, survival, and aquatic vertebrate model analysis. Many studies have delivered new therapeutic metabolites for disease prevention, and toxicity with limited success in aquatic zebrafish model (Lu et al., 2016; Kooij et al., 2020). We took advantages from state-of-the-art NMR infrastructure with high reproducibility, quotative capacity, and robustness. This analysis precludes the assignment of ^1^H-edited NMR-detected fish metabolites.

When analyse each metabolite, we found a pattern of changes in the metabolic intricate of zebrafish. The most remarkable alteration found in our analysis was reduction in the abundance of metabolites that contribute directly or indirectly to metabolic reprograming in zebrafish (Huang et al., 2016). Interestingly, MSEA highlighted the enrichment in glycolysis/gluconeogenesis in zebrafish, an observation in our recent report on rewiring an energy metabolism in various cellular environments (Huang et al., 2016; Lu et al., 2016). In the cellular environments, nanoparticles reported to induce structural transformations within cell such as secondary lysosome formation (Shvedova et al., 2003; Monteiro-Riviere et al., 2005; Yuan et al., 2012). The perturbed NADPH status could inhibit the glutathione cycle (GSH/GSSG) which results in induction of oxidative stress (Gluick and Yadav, 2003).

Remarkably, we found that SWCNT induced major metabolic pathways in zebrafish metabolism and are quantified by MESA investigations. The creatine and creatine phosphate are modified that may fail to serve as energy in cell. Here, purine metabolism might be disconnected from energy metabolisms such as Krebs cycle and glycolysis (Laíns et al., 2019). The enriched metabolic functions with SWCNT treatment have bordered and shown to clearly connected to cell death and survival, proliferation, phenotypic expression, and cellular microenvironment.

According to these data, we quantified a set of metabolic pathways, glycolysis/gluconeogenesis, primary bile acid biosynthesis, phenylalanine metabolism, phenylalanine, tyrosine, and tryptophan biosynthesis, taurine and hypotaurine metabolism, ether lipid metabolism, citrate cycle might play important role in 10 mg/L of SWCNT exposure in zebrafish. The cysteine and methionine metabolism, ether lipid metabolism, pantothenate and CoA biosynthesis, taurine and hypotaurine metabolism, primary bile acid biosynthesis, glycine, serine and threonine metabolism are modified in 50 mg/L of SWCNT treatment in zebrafish. Finaly, we extended the quantification of metabolic pathway in 100 mg/L of SWCNT treatment. Purine metabolism, glycolysis/gluconeogenesis, taurine and hypotaurine metabolism, citrate cycle and primary bile acid biosynthesis are impacted.

## Conclusion

This study convincingly demonstrated the impacts of metabolites caused by SWCNT exposure in zebrafish for the first time at the omics scale. According to our findings based on ^1^H-NMR technique, SWCNT exposure caused significant phenotypic changes in metabolites. ^1^H-NMR offers significant benefits to the metabolomics field via the detection of metabolites integral to critical cellular metabolism and cell signaling. Malate, oxalacetate, phenylaniline, taurine, sn-glycero-3-phosphate, glycine, N-acetyl mate, lactate, ATP, AMP, valine, pyruvate, ADP, serine, niacinamide are significantly impacted by SWCNT. We believe that these metabolome alterations and metabolic pathways in zebrafish, such as energy, amino acid, and nucleotide metabolisms, may contribute in the development of future treatments that target diseases, especially as SWCNT exposure becomes more important as a pharmacological target. However, more investigations are necessary to better understand the SWCNT exposure and underlying mechanisms.

## Data Availability

The original contributions presented in the study are included in the article/Supplementary Material, further inquiries can be directed to the corresponding authors.
