# Supplementary material for: Carbon Nanotubes Induce Metabolomic Profile Disturbances in Zebrafish: NMR-Based Metabolomics Platform
Source: Front Mol Biosci. 2021 Jul 2;8:688827. doi: 10.3389/fmolb.2021.688827 (PMC8283261; doi:10.3389/fmolb.2021.688827)
Supplement: Supplementary file 1 [file DataSheet1.docx]

**SUPPLEMENTARY MATERIAL**

**Carbon Nanotubes induce Metabolomic profile Disturbances in Zebrafish: NMR-based Metabolomics platform**

Raja Ganesan^1,2^, Prabhakaran Vasantha-Srinivasan^4^, Deepa Rani Sadhasivam^5^, Raghunandhakumar Subramanian^3^, Selvaraj Vimalraj ^6,3*^ and Ki Tae Suk^1*^

^1^Institute for Liver and Digestive Diseases, Hallym University, Chuncheon 24252, Republic of Korea.

^2^Department of Biological Sciences, Pusan National University, Busan, 46241, Republic of Korea.

^3^Department of Pharmacology, Saveetha Dental College and Hospital, Saveetha Institute of Medical and Technical Sciences (SIMATS), Saveetha University, Chennai 600 077, Tamil Nadu, India.

^4^Department of Biotechnology, St. Peter's Institute of Higher Education and Research, Avadi, 600 054, Chennai, Tamil Nadu, India.

^5^PG & Research Department of Zoology, Ethiraj College for Women, Chennai, Tamil Nadu, India.

^6^Center for Biotechnology, Anna University, Chennai, 600 025, India.

*** Correspondence:**

[vimalr50@gmail.com](mailto:vimalr50@gmail.com) (S.V)

[ktsuk@hallym.ac.kr](mailto:ktsuk@hallym.ac.kr) (K.T.S)

**Supplementary Figure S1.**

**
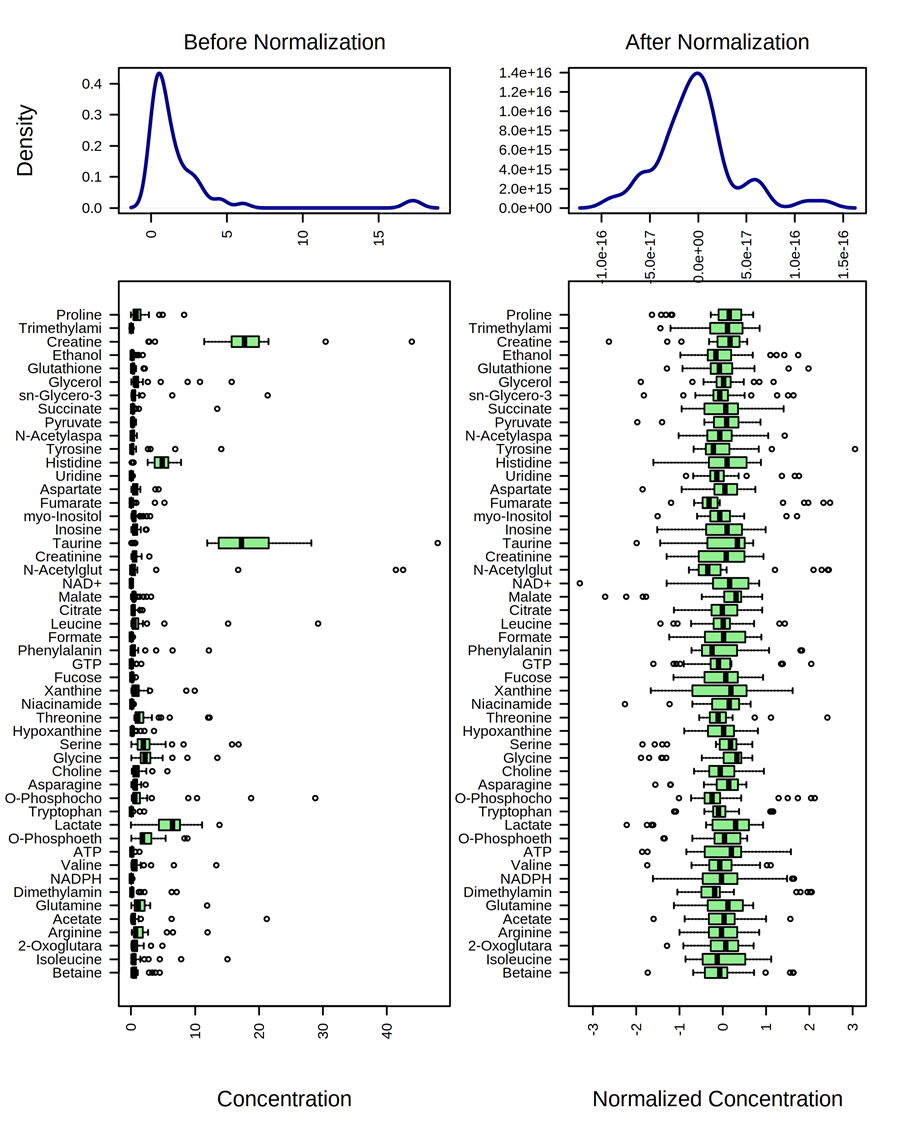
**

**Supplementary Figure S1|** *Pareto Scaling-*based Kernel density plots and Box plots analysis before and after normalization with SWCNT exposures. The box plots show at most quantified metabolites and the density plots are based on all samples. The graph has been summarized the distribution of input metabolome data values before and after normalization. The box plots show the concentration distributions of individual spectra bins, whereas the complete concentration distribution based on kernel density (*Pareto Scaling*) estimation.

**Supplementary Figure S2| (A)**

**
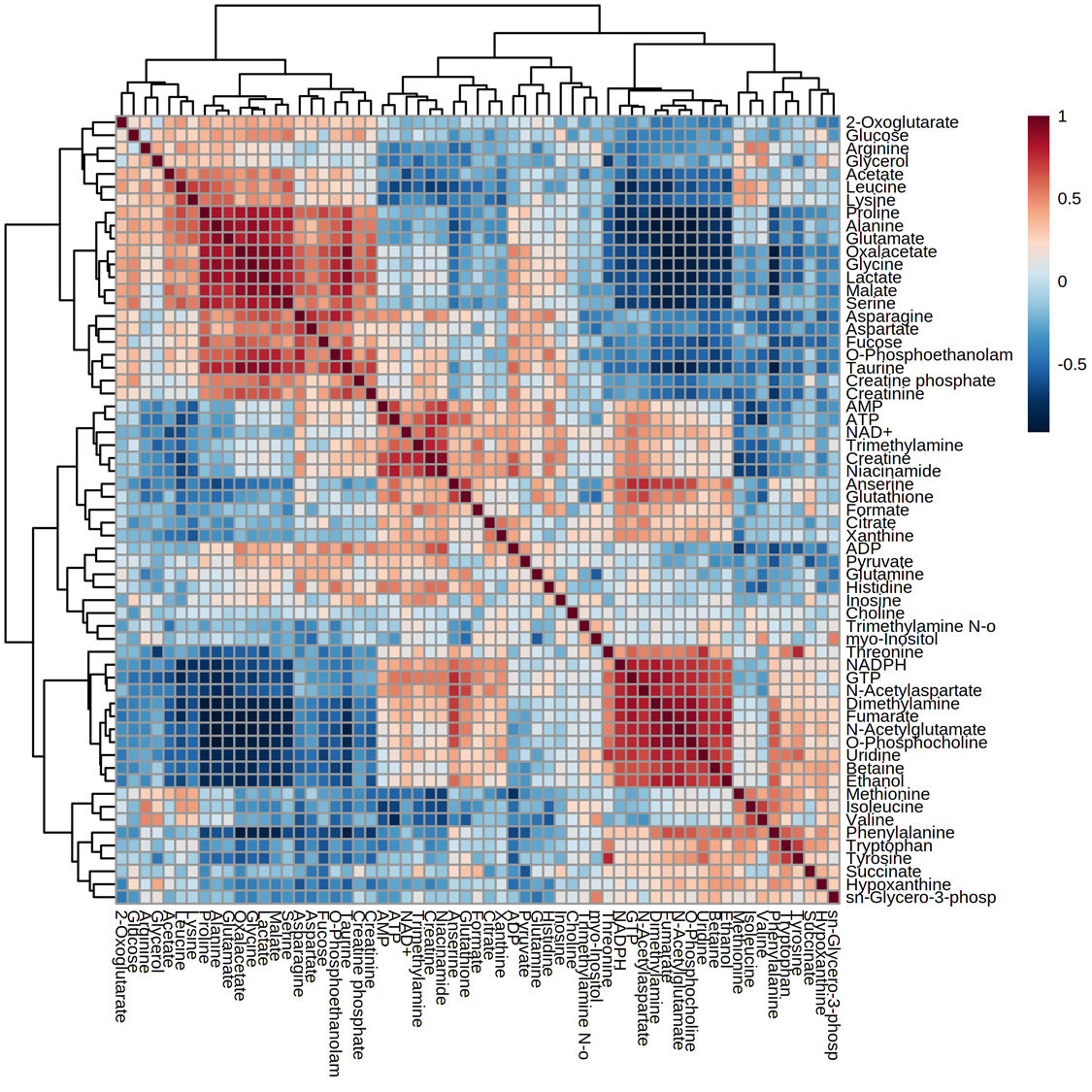
**

**(B)**

**
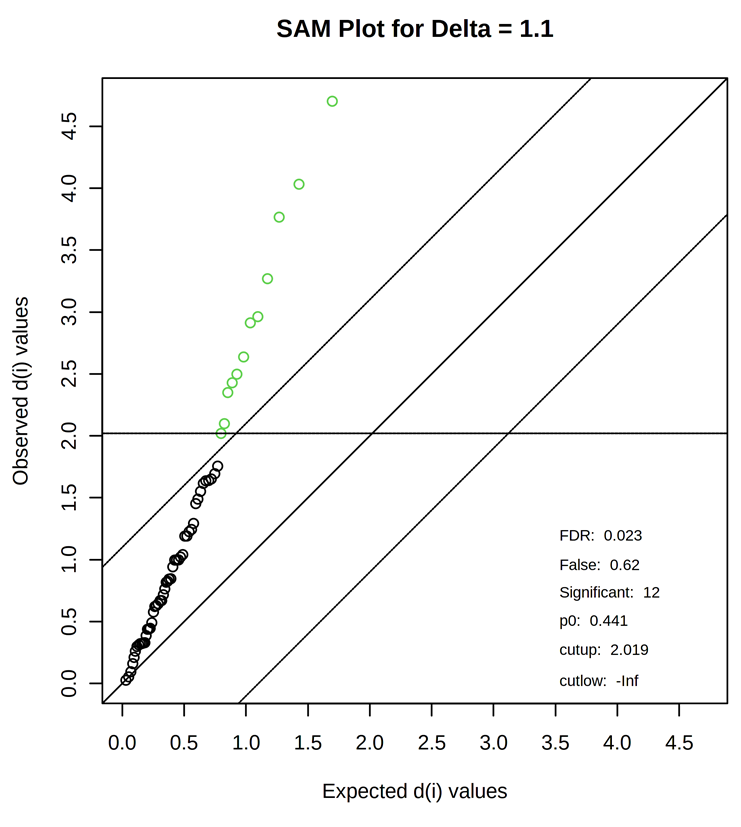
**

**(C)**

**
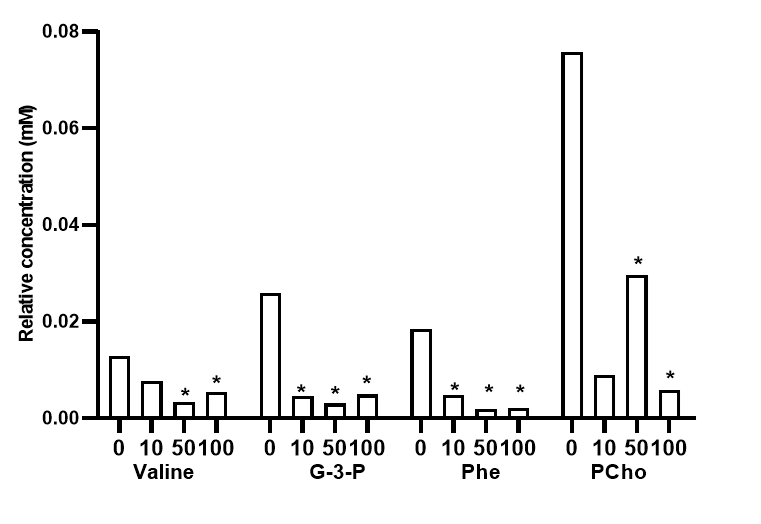
**

**Supplementary Figure S2| (A)** Correlogram of metabolites after SWCNT treatment. Data of the relative concentration of metabolites were normalized and analyzed using Spearman rank correlation analysis (*r*- values) within MetaboAnalyst (v 5.0). Each coloured cell on the map indicates the correlation coefficient, with the scale code shown on the top right corner (red and blue colours mean positive and negative correlations, respectively). **(B)** Significance analysis of microarrays (SAM) shows 12 significant metabolites has been identified with SWCNT applied metabolites. **(C)** From the SAM plot significances, very few metabolites such as valine, G-3-P, sn-Glycero-3-phosphocholine; Phe, Phenylalanine; PCho, O-Phosphocholine has been significantly identified from SWCNT exposures.

**Supplementary Figure S3| (A)**

**
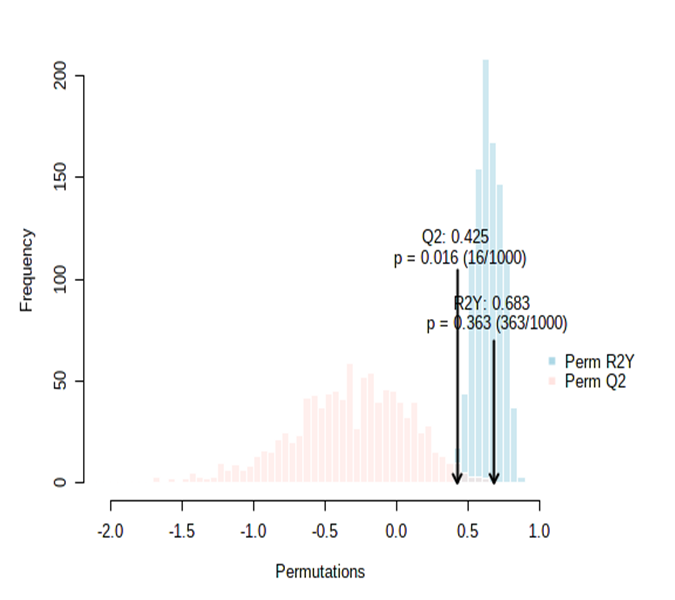
**

**(B)**

**
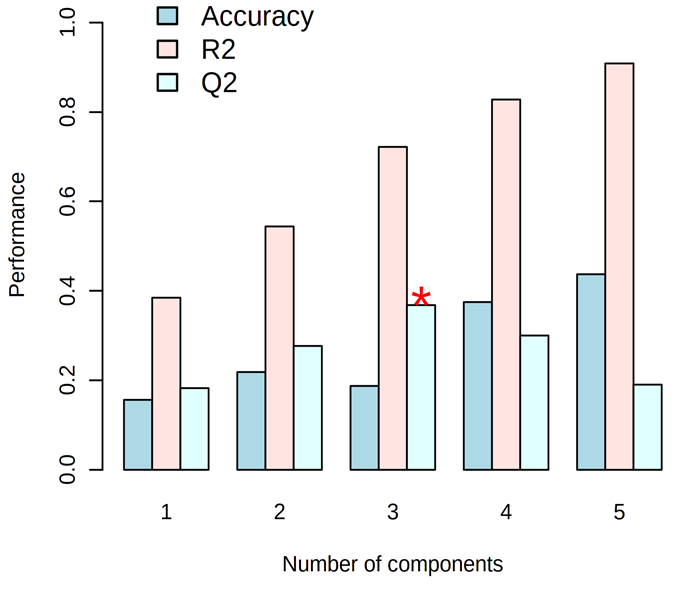
**

**Supplementary Figure S3| (A)** Permutation test; Select test statistic: Separation distance (B/W), set permutation numbers:1000 p<0.016; Q^2^: 0.425; R^2^Y: 0.683. **(B)** An accuracy and sample performance has been identified with SWCNT applied.

**Supplementary Table S1|** The metabolic pathways enrichment analysis performed and quantified the significant pathways in 10 mg/L of SWCNT in zebrafish.

|  | **Metabolite Set** | **Total** | **Hits** | **Statistic** | **P value** | **Holm P** | **FDR** |
| --- | --- | --- | --- | --- | --- | --- | --- |
| ﻿ | Glycolysis / Gluconeogenesis | 26 | 5 | 34.389 | 0.0044595 | 0.21406 | 0.03677 |
| ﻿ | Primary bile acid biosynthesis | 46 | 2 | 42.193 | 0.0046949 | 0.22066 | 0.03677 |
| ﻿ | Phenylalanine metabolism | 10 | 2 | 35.472 | 0.0063845 | 0.29368 | 0.03677 |
| ﻿ | Phenylalanine, tyrosine and tryptophan biosynthesis | 4 | 2 | 35.472 | 0.0063845 | 0.29368 | 0.03677 |
| ﻿ | Taurine and hypotaurine metabolism | 8 | 1 | 40.53 | 0.0080069 | 0.3523 | 0.03677 |
| ﻿ | Ether lipid metabolism | 20 | 1 | 40.277 | 0.008269 | 0.35557 | 0.03677 |
| ﻿ | Citrate cycle (TCA cycle) | 20 | 6 | 27.132 | 0.0084902 | 0.35659 | 0.03677 |
| ﻿ | Aminoacyl-tRNA biosynthesis | 48 | 19 | 22.185 | 0.0093624 | 0.38386 | 0.03677 |
| ﻿ | Glycine, serine and threonine metabolism | 33 | 7 | 24.704 | 0.0094578 | 0.38386 | 0.03677 |
| ﻿ | Glyoxylate and dicarboxylate metabolism | 32 | 9 | 25.209 | 0.0098639 | 0.38469 | 0.03677 |
| ﻿ | Alanine, aspartate and glutamate metabolism | 28 | 12 | 24.637 | 0.010524 | 0.3999 | 0.03677 |
| ﻿ | Porphyrin and chlorophyll metabolism | 30 | 2 | 36.612 | 0.011103 | 0.41081 | 0.03677 |
| ﻿ | Fructose and mannose metabolism | 20 | 1 | 37.646 | 0.011475 | 0.4131 | 0.03677 |
| ﻿ | Amino sugar and nucleotide sugar metabolism | 37 | 1 | 37.646 | 0.011475 | 0.4131 | 0.03677 |
| ﻿ | Pyruvate metabolism | 22 | 5 | 30.434 | 0.011491 | 0.4131 | 0.03677 |
| ﻿ | Glycerophospholipid metabolism | 36 | 4 | 24.232 | 0.015316 | 0.50543 | 0.043263 |
| ﻿ | Tyrosine metabolism | 42 | 3 | 23.957 | 0.015322 | 0.50543 | 0.043263 |
| ﻿ | Glutathione metabolism | 28 | 3 | 26.144 | 0.017413 | 0.53981 | 0.046435 |
| ﻿ | Butanoate metabolism | 15 | 3 | 20.148 | 0.020123 | 0.60369 | 0.048197 |
| ﻿ | Histidine metabolism | 16 | 3 | 20.451 | 0.020531 | 0.60369 | 0.048197 |
| ﻿ | Cysteine and methionine metabolism | 33 | 3 | 19.916 | 0.021209 | 0.60369 | 0.048197 |
| ﻿ | Sphingolipid metabolism | 21 | 2 | 28.111 | 0.022817 | 0.61605 | 0.048197 |
| ﻿ | Pantothenate and CoA biosynthesis | 19 | 2 | 25.653 | 0.023095 | 0.61605 | 0.048197 |
| ﻿ | Arginine biosynthesis | 14 | 7 | 22.396 | 0.029712 | 0.74281 | 0.059425 |
| ﻿ | Arginine and proline metabolism | 38 | 6 | 16.945 | 0.033628 | 0.80706 | 0.064565 |
| ﻿ | Selenocompound metabolism | 20 | 1 | 24.616 | 0.050625 | 1.0 | 0.093461 |
| ﻿ | beta-Alanine metabolism | 21 | 2 | 17.999 | 0.057748 | 1.0 | 0.10091 |
| ﻿ | D-Glutamine and D-glutamate metabolism | 6 | 5 | 16.695 | 0.058866 | 1.0 | 0.10091 |
| ﻿ | Ubiquinone and other terpenoid-quinone biosynthesis | 9 | 1 | 22.126 | 0.065953 | 1.0 | 0.10916 |
| ﻿ | Valine, leucine and isoleucine biosynthesis | 8 | 4 | 15.358 | 0.080518 | 1.0 | 0.12883 |
| ﻿ | Nitrogen metabolism | 6 | 2 | 15.575 | 0.090373 | 1.0 | 0.13993 |
| ﻿ | Valine, leucine and isoleucine degradation | 40 | 3 | 14.009 | 0.11616 | 1.0 | 0.17424 |
| ﻿ | Starch and sucrose metabolism | 18 | 1 | 14.201 | 0.15022 | 1.0 | 0.21207 |
| ﻿ | Neomycin, kanamycin and gentamicin biosynthesis | 2 | 1 | 14.201 | 0.15022 | 1.0 | 0.21207 |
| ﻿ | Phosphonate and phosphinate metabolism | 6 | 1 | 12.869 | 0.17242 | 1.0 | 0.23646 |
| ﻿ | Tryptophan metabolism | 41 | 1 | 11.801 | 0.19268 | 1.0 | 0.2569 |
| ﻿ | Propanoate metabolism | 23 | 1 | 11.444 | 0.2 | 1.0 | 0.25946 |
| ﻿ | Folate biosynthesis | 27 | 1 | 10.958 | 0.21043 | 1.0 | 0.26581 |
| ﻿ | Pyrimidine metabolism | 39 | 2 | 8.4713 | 0.27574 | 1.0 | 0.33938 |
| ﻿ | Nicotinate and nicotinamide metabolism | 15 | 3 | 8.1334 | 0.28841 | 1.0 | 0.34609 |
| ﻿ | Purine metabolism | 65 | 8 | 7.6902 | 0.29728 | 1.0 | 0.34803 |
| ﻿ | Galactose metabolism | 27 | 3 | 5.469 | 0.49187 | 1.0 | 0.56214 |
| ﻿ | Glycerolipid metabolism | 16 | 1 | 1.9838 | 0.60286 | 1.0 | 0.63487 |
| ﻿ | Ascorbate and aldarate metabolism | 8 | 1 | 1.9241 | 0.60842 | 1.0 | 0.63487 |
| ﻿ | Inositol phosphate metabolism | 30 | 1 | 1.9241 | 0.60842 | 1.0 | 0.63487 |
| ﻿ | Phosphatidylinositol signaling system | 28 | 1 | 1.9241 | 0.60842 | 1.0 | 0.63487 |
| ﻿ | Lysine degradation | 25 | 1 | 0.77303 | 0.7461 | 1.0 | 0.7461 |
| ﻿ | Biotin metabolism | 10 | 1 | 0.77303 | 0.7461 | 1.0 | 0.7461 |

**Supplementary Table S2|** The metabolic pathways enrichment analysis performed and quantified the significant pathways in 50 mg/L of SWCNT in zebrafish.

|  | **Metabolite Set** | **Total** | **Hits** | **Statistic** | **P value** | **Holm P** | **FDR** |
| --- | --- | --- | --- | --- | --- | --- | --- |
| ﻿ | Cysteine and methionine metabolism | 33 | 3 | 31.216 | 0.001432 | 0.068738 | 0.068738 |
| ﻿ | Ether lipid metabolism | 20 | 1 | 44.379 | 0.0048387 | 0.22742 | 0.082297 |
| ﻿ | Pantothenate and CoA biosynthesis | 19 | 2 | 36.902 | 0.0051436 | 0.23661 | 0.082297 |
| ﻿ | Taurine and hypotaurine metabolism | 8 | 1 | 35.649 | 0.014609 | 0.65739 | 0.1011 |
| ﻿ | Primary bile acid biosynthesis | 46 | 2 | 33.677 | 0.015848 | 0.69729 | 0.1011 |
| ﻿ | Glycine, serine and threonine metabolism | 33 | 7 | 20.956 | 0.016588 | 0.71329 | 0.1011 |
| ﻿ | Valine, leucine and isoleucine degradation | 40 | 3 | 23.992 | 0.017525 | 0.73604 | 0.1011 |
| ﻿ | Aminoacyl-tRNA biosynthesis | 48 | 19 | 18.022 | 0.019832 | 0.8131 | 0.1011 |
| ﻿ | Phenylalanine metabolism | 10 | 2 | 24.353 | 0.024225 | 0.96901 | 0.1011 |
| ﻿ | Phenylalanine, tyrosine and tryptophan biosynthesis | 4 | 2 | 24.353 | 0.024225 | 0.96901 | 0.1011 |
| ﻿ | Glyoxylate and dicarboxylate metabolism | 32 | 9 | 21.496 | 0.024682 | 0.96901 | 0.1011 |
| ﻿ | Glycolysis / Gluconeogenesis | 26 | 5 | 24.55 | 0.025507 | 0.96901 | 0.1011 |
| ﻿ | Sphingolipid metabolism | 21 | 2 | 26.036 | 0.028618 | 1.0 | 0.1011 |
| ﻿ | Valine, leucine and isoleucine biosynthesis | 8 | 4 | 18.27 | 0.02963 | 1.0 | 0.1011 |
| ﻿ | Glycerophospholipid metabolism | 36 | 4 | 19.031 | 0.031594 | 1.0 | 0.1011 |
| ﻿ | Citrate cycle (TCA cycle) | 20 | 6 | 19.397 | 0.039946 | 1.0 | 0.11984 |
| ﻿ | Purine metabolism | 65 | 8 | 14.835 | 0.047172 | 1.0 | 0.12675 |
| ﻿ | Pyruvate metabolism | 22 | 5 | 20.837 | 0.048355 | 1.0 | 0.12675 |
| ﻿ | Alanine, aspartate and glutamate metabolism | 28 | 12 | 17.146 | 0.050172 | 1.0 | 0.12675 |
| ﻿ | Porphyrin and chlorophyll metabolism | 30 | 2 | 21.023 | 0.066373 | 1.0 | 0.15568 |
| ﻿ | Starch and sucrose metabolism | 18 | 1 | 21.378 | 0.071355 | 1.0 | 0.15568 |
| ﻿ | Neomycin, kanamycin and gentamicin biosynthesis | 2 | 1 | 21.378 | 0.071355 | 1.0 | 0.15568 |
| ﻿ | Arginine and proline metabolism | 38 | 6 | 13.499 | 0.092307 | 1.0 | 0.19264 |
| ﻿ | Glutathione metabolism | 28 | 3 | 14.9 | 0.10656 | 1.0 | 0.21312 |
| ﻿ | Nicotinate and nicotinamide metabolism | 15 | 3 | 13.717 | 0.12033 | 1.0 | 0.22374 |
| ﻿ | Arginine biosynthesis | 14 | 7 | 13.68 | 0.12119 | 1.0 | 0.22374 |
| ﻿ | beta-Alanine metabolism | 21 | 2 | 13.364 | 0.13337 | 1.0 | 0.22928 |
| ﻿ | Tyrosine metabolism | 42 | 3 | 12.124 | 0.13888 | 1.0 | 0.22928 |
| ﻿ | Galactose metabolism | 27 | 3 | 12.117 | 0.14153 | 1.0 | 0.22928 |
| ﻿ | D-Glutamine and D-glutamate metabolism | 6 | 5 | 12.145 | 0.1433 | 1.0 | 0.22928 |
| ﻿ | Tryptophan metabolism | 41 | 1 | 12.051 | 0.18771 | 1.0 | 0.29065 |
| ﻿ | Histidine metabolism | 16 | 3 | 10.39 | 0.19896 | 1.0 | 0.29195 |
| ﻿ | Nitrogen metabolism | 6 | 2 | 10.888 | 0.20072 | 1.0 | 0.29195 |
| ﻿ | Fructose and mannose metabolism | 20 | 1 | 10.799 | 0.21398 | 1.0 | 0.29345 |
| ﻿ | Amino sugar and nucleotide sugar metabolism | 37 | 1 | 10.799 | 0.21398 | 1.0 | 0.29345 |
| ﻿ | Glycerolipid metabolism | 16 | 1 | 10.435 | 0.22233 | 1.0 | 0.29644 |
| ﻿ | Pyrimidine metabolism | 39 | 2 | 9.5343 | 0.24614 | 1.0 | 0.31932 |
| ﻿ | Selenocompound metabolism | 20 | 1 | 7.8089 | 0.29456 | 1.0 | 0.34211 |
| ﻿ | Ascorbate and aldarate metabolism | 8 | 1 | 7.7326 | 0.29703 | 1.0 | 0.34211 |
| ﻿ | Inositol phosphate metabolism | 30 | 1 | 7.7326 | 0.29703 | 1.0 | 0.34211 |
| ﻿ | Phosphatidylinositol signaling system | 28 | 1 | 7.7326 | 0.29703 | 1.0 | 0.34211 |
| ﻿ | Butanoate metabolism | 15 | 3 | 8.3093 | 0.29935 | 1.0 | 0.34211 |
| ﻿ | Propanoate metabolism | 23 | 1 | 4.2576 | 0.44325 | 1.0 | 0.49479 |
| ﻿ | Phosphonate and phosphinate metabolism | 6 | 1 | 3.2129 | 0.50654 | 1.0 | 0.55259 |
| ﻿ | Folate biosynthesis | 27 | 1 | 1.7472 | 0.62556 | 1.0 | 0.64603 |
| ﻿ | Lysine degradation | 25 | 1 | 1.6778 | 0.63257 | 1.0 | 0.64603 |
| ﻿ | Biotin metabolism | 10 | 1 | 1.6778 | 0.63257 | 1.0 | 0.64603 |
| ﻿ | Ubiquinone and other terpenoid-quinone biosynthesis | 9 | 1 | 1.5411 | 0.64691 | 1.0 | 0.64691 |

**Supplementary Table S3|** The metabolic pathways enrichment analysis performed and quantified the significant pathways in 100 mg/L of SWCNT in zebrafish.

|  | **Metabolite Set** | **Total** | **Hits** | **Statistic** | **P value** | **Holm P** | **FDR** |
| --- | --- | --- | --- | --- | --- | --- | --- |
| ﻿ | Purine metabolism | 65 | 8 | 22.149 | 0.0016797 | 0.080627 | 0.03003 |
| ﻿ | Glycolysis / Gluconeogenesis | 26 | 5 | 38.266 | 0.0030227 | 0.14207 | 0.03003 |
| ﻿ | Taurine and hypotaurine metabolism | 8 | 1 | 47.741 | 0.0030385 | 0.14207 | 0.03003 |
| ﻿ | Citrate cycle (TCA cycle) | 20 | 6 | 33.231 | 0.0036814 | 0.16566 | 0.03003 |
| ﻿ | Primary bile acid biosynthesis | 46 | 2 | 42.858 | 0.0048362 | 0.21279 | 0.03003 |
| ﻿ | Tyrosine metabolism | 42 | 3 | 28.773 | 0.0053966 | 0.23205 | 0.03003 |
| ﻿ | Glycine, serine and threonine metabolism | 33 | 7 | 26.979 | 0.0054341 | 0.23205 | 0.03003 |
| ﻿ | Phenylalanine metabolism | 10 | 2 | 36.547 | 0.0056305 | 0.23205 | 0.03003 |
| ﻿ | Phenylalanine, tyrosine and tryptophan biosynthesis | 4 | 2 | 36.547 | 0.0056305 | 0.23205 | 0.03003 |
| ﻿ | Alanine, aspartate and glutamate metabolism | 28 | 12 | 28.265 | 0.0094821 | 0.3698 | 0.043146 |
| ﻿ | Pyruvate metabolism | 22 | 5 | 33.272 | 0.0098876 | 0.37573 | 0.043146 |
| ﻿ | beta-Alanine metabolism | 21 | 2 | 27.696 | 0.011175 | 0.41347 | 0.044699 |
| ﻿ | Glyoxylate and dicarboxylate metabolism | 32 | 9 | 27.523 | 0.01375 | 0.49502 | 0.050771 |
| ﻿ | Cysteine and methionine metabolism | 33 | 3 | 23.602 | 0.015487 | 0.54205 | 0.053099 |
| ﻿ | Histidine metabolism | 16 | 3 | 25.255 | 0.016896 | 0.57446 | 0.053986 |
| ﻿ | Glycerophospholipid metabolism | 36 | 4 | 22.587 | 0.018918 | 0.62428 | 0.053986 |
| ﻿ | Arginine biosynthesis | 14 | 7 | 27.142 | 0.01912 | 0.62428 | 0.053986 |
| ﻿ | Butanoate metabolism | 15 | 3 | 22.473 | 0.021452 | 0.66501 | 0.055844 |
| ﻿ | Porphyrin and chlorophyll metabolism | 30 | 2 | 31.105 | 0.02262 | 0.67861 | 0.055844 |
| ﻿ | Aminoacyl-tRNA biosynthesis | 48 | 19 | 19.872 | 0.024479 | 0.7099 | 0.055844 |
| ﻿ | Glutathione metabolism | 28 | 3 | 25.081 | 0.026471 | 0.7412 | 0.055844 |
| ﻿ | Fructose and mannose metabolism | 20 | 1 | 30.076 | 0.027834 | 0.75152 | 0.055844 |
| ﻿ | Amino sugar and nucleotide sugar metabolism | 37 | 1 | 30.076 | 0.027834 | 0.75152 | 0.055844 |
| ﻿ | Ether lipid metabolism | 20 | 1 | 30.045 | 0.027929 | 0.75152 | 0.055844 |
| ﻿ | Pantothenate and CoA biosynthesis | 19 | 2 | 20.988 | 0.029085 | 0.75152 | 0.055844 |
| ﻿ | Sphingolipid metabolism | 21 | 2 | 24.984 | 0.041302 | 0.94995 | 0.07625 |
| ﻿ | Arginine and proline metabolism | 38 | 6 | 18.125 | 0.045647 | 1.0 | 0.08115 |
| ﻿ | D-Glutamine and D-glutamate metabolism | 6 | 5 | 18.84 | 0.056403 | 1.0 | 0.096691 |
| ﻿ | Propanoate metabolism | 23 | 1 | 22.754 | 0.061721 | 1.0 | 0.10216 |
| ﻿ | Pyrimidine metabolism | 39 | 2 | 19.376 | 0.067263 | 1.0 | 0.10762 |
| ﻿ | Nitrogen metabolism | 6 | 2 | 17.92 | 0.072722 | 1.0 | 0.1126 |
| ﻿ | Phosphonate and phosphinate metabolism | 6 | 1 | 18.394 | 0.09739 | 1.0 | 0.14427 |
| ﻿ | Ubiquinone and other terpenoid-quinone biosynthesis | 9 | 1 | 18.218 | 0.099184 | 1.0 | 0.14427 |
| ﻿ | Folate biosynthesis | 27 | 1 | 16.855 | 0.11421 | 1.0 | 0.16124 |
| ﻿ | Tryptophan metabolism | 41 | 1 | 15.548 | 0.13071 | 1.0 | 0.17926 |
| ﻿ | Nicotinate and nicotinamide metabolism | 15 | 3 | 12.03 | 0.15015 | 1.0 | 0.20021 |
| ﻿ | Selenocompound metabolism | 20 | 1 | 13.41 | 0.16302 | 1.0 | 0.21149 |
| ﻿ | Valine, leucine and isoleucine degradation | 40 | 3 | 9.7232 | 0.22617 | 1.0 | 0.28569 |
| ﻿ | Valine, leucine and isoleucine biosynthesis | 8 | 4 | 8.3255 | 0.27389 | 1.0 | 0.3371 |
| ﻿ | Glycerolipid metabolism | 16 | 1 | 4.491 | 0.43074 | 1.0 | 0.51689 |
| ﻿ | Ascorbate and aldarate metabolism | 8 | 1 | 1.5035 | 0.65098 | 1.0 | 0.72668 |
| ﻿ | Inositol phosphate metabolism | 30 | 1 | 1.5035 | 0.65098 | 1.0 | 0.72668 |
| ﻿ | Phosphatidylinositol signaling system | 28 | 1 | 1.5035 | 0.65098 | 1.0 | 0.72668 |
| ﻿ | Starch and sucrose metabolism | 18 | 1 | 0.70037 | 0.75798 | 1.0 | 0.80851 |
| ﻿ | Neomycin, kanamycin and gentamicin biosynthesis | 2 | 1 | 0.70037 | 0.75798 | 1.0 | 0.80851 |
| ﻿ | Galactose metabolism | 27 | 3 | 2.1309 | 0.79953 | 1.0 | 0.8343 |
| ﻿ | Lysine degradation | 25 | 1 | 0.20631 | 0.86734 | 1.0 | 0.86734 |
| ﻿ | Biotin metabolism | 10 | 1 | 0.20631 | 0.86734 | 1.0 | 0.86734 |
